# Modeling the non-integer dynamics of a vector-borne infection with nonlocal and nonsingular kernel

**DOI:** 10.1038/s41598-025-90182-1

**Published:** 2025-02-20

**Authors:** Nekmat Ullah, Zahir Shah, Rashid Jan, Narcisa Vrinceanu, Muhammad Farhan, Elisabeta Antonescu

**Affiliations:** 1 Department of Mathematical Sciences, University of Lakki Marwat, Lakki Marwat, KPK 28420 Pakistan; 2 Institute of Energy Infrastructure (IEI), Department of Civil Engineering, College of Engineering, Universiti Tenaga Nasional (UNITEN), Putrajaya Campus, Jalan IKRAM-UNITEN, 43000 Kajang, Selangor Malaysia; 3 Mathematics Research Center, Near East University TRNC, Mersin 10, 99138 Nicosia, Turkey; 4 Department of Mathematics, College of Science, King Saud University, P.O. Box 2455, 11451 Riyadh, Saudi Arabia; 5 School of Mathematical Science, Yangzhou University, Yangzhou, 225002 China; 6 Preclinical Department Faculty of Medicine, Lucian Blaga University of Sibiu, Sibiu, Romania

**Keywords:** Fractional calculus, Vector-borne infection, Mathematical model, Existence theory, Fixed-point theorem, Numerical analysis, Biological techniques, Mathematics and computing

## Abstract

Vector-borne infections impose a significant burden on global health systems and economies due to their widespread impact and the substantial resources required for prevention, control, and treatment efforts. In this work, we formulate a mathematical model for the transmission dynamics of a vector-borne infection with the effect of vaccination through the Atangana-Baleanu derivative. The solutions of the model are positive and bounded for positive initial values of the state variable. We presented the basic concept and theory of fractional calculus for the analysis of the model. We determine the threshold parameter, denoted by $\mathcal {R}_0$, using the next-generation matrix method. The local asymptotic stability of the system at the disease-free equilibrium is analyzed. To establish the existence of solutions for the proposed model, we employ fixed-point theory. A numerical scheme is developed to visualize the system’s dynamical behavior under varying input parameters. Numerical simulations are conducted to illustrate how these parameters influence the dynamics of the system. The results highlight key factors affecting the transmission and control of vector-borne diseases, offering insights into strategies for prevention and mitigation.

## Introduction

Vector-borne diseases (VBDs) include malaria, dengue fever, Zika virus, and Chikungunya, all of which are major health issues in the world especially in the tropics and subtropics. Malaria alone, for example, caused 247 million cases and 619,000 deaths in 2021-mainly in Africa. Changing climate patterns, urbanization as well as global mobility have made these diseases persist despite various preventive strategies which are put in place to fight them. Infections that are spread by vectors are illnesses passed on to people and animals via insects like mosquitoes, ticks, flies, and fleas^1^. These insects carry germs like viruses, bacteria, and parasites which they introduce into new hosts during meals taken from their blood^2^. Vector-borne diseases have been a significant issue in public health since ancient times. Malignant fevers like malaria and typhus left Greece and Rome devastated, as they noted down back then. It was not until the late 19th century and early 20th century that scientists learned of the bacteria responsible for such diseases. A turning point in vector-borne disease research occurred leading to substantial advancements in preventive strategies; however, vectors are still major health challenges globally due to factors like climate change, urbanization, and human migration, which hastened their spread^3^.

Vector-borne diseases are very big health problems globally. If we take a look at the World Health Organization (WHO), they estimate that there are more than 17% of all infections that are posed by vector-borne diseases leading to over 700,000 deaths every year. Among the most significant concern is malaria, with approximately 219 million cases occurring and over 400,000 deaths yearly mostly in children below 5 years old living in sub-Saharan Africa^4^. As social and economic factors are now included as important influencing variables on malaria transmission, the new model has been developed. In fact, malaria really digs deep into the health of countries around the world, most severely infecting millions of people, especially younger children in tropical areas. Unlike earlier models, this one incorporates family conditions and household conditions, such that these models are better equipped to describe the influence of such conditions on both disease transmission and disease severity^5^. The Marburg virus is an infectious disease; due to this infection more deaths are reported. It even causes more cases in places where healthcare is really much more difficult to contain. The cause of this surge is entirely delaying action on speedy interventions to fight against the virus^6^. Dengue fever threatens an estimated 3.9 billion people across 129 nations around the globe with around 96 million symptomatic cases and about 40,000 related deaths each year^7^. Climate change has a direct impact on these ailments due to its effect on the ecosystem of vectors such as their distribution patterns, physiology or behavior which implies that it will lead to higher chances of transmission and more severe epidemics^8^.

Diseases borne by significant vectors include malaria, dengue fever, Zika virus, Lyme disease, and chikungunya. The weight of these infections is particularly high in tropical and subtropical areas where environmental conditions favor the proliferation of vectors. Climate change, urbanization, and increased global travel worsen the situation^9^. To effectively control and prevent them, an integrated approach that includes vector control measures, public health education, vaccination programs as well as strong healthcare systems, is essential^10^. The comprehension that diseases spread via vectors and the influence of different factors on that spread is of utmost importance for the creation of specific measures and mitigation of their global health effects^11^. One of the major components of understanding infectious diseases, predicting epidemics, assessing control efforts, guiding treatment policies, stimulating research as well as improving education, and raising public awareness is through mathematical models^12^. They are integral to the global efforts in controlling and preventing these significant public health threats^13^. In addition, deforestation has a significant effect on transmission and spread through breeding areas, healthcare services access, movement and migration over time as well as population density in specific regions. Understanding how these variables work together is important for devising effective prevention and control strategies for such illnesses. In this perspective, vaccination is modeled as a compartmental type model assuming that the reduced probability of infection is with vaccinated individuals. The effects of vaccination on transmission dynamics are shown through the use of the Atangana-Baleanu derivative to allow a more realistic representation of the effects of vaccination on the spread of disease^14^.

Fractional differential operators are increasingly being applied in different scientific and engineering fields, particularly in the area of mathematical modeling, which has emerged recently^17^. Within this perspective, it is crucial that we focus on Atangana-Baleanu fractional differential operators since they factor in the intricacy of binding illnesses such as Dengue and Zika^18^. Furthermore, fractional differential operators have found applications in addressing complex real-life issues like drug trafficking, corruption, and new chaotic systems apart from their use in epidemiology^19^. These operators can be distinguished from traditional integer order operators through the employment of non-locality and long-term memory effects by the use of the Mittage-Leffler function as their kernel^20^. They have been employed in several epidemiological models, such as Covid-19 model, HPV model, and syphilis model, which demonstrate how closely they capture disease behaviors^21^. Measles is a leading cause of child deaths, killing about 142,300 people yearly, even though there’s a safe vaccine. Cases have gone up recently, especially in young children and adults with weak immune systems. The main reason for this rise is fewer people getting vaccinated^22^. It is possible to qualitatively and quantitatively analyze vector-borne infections through a fractional framework that makes use of non-integer derivatives to model their dynamics accurately^23^. This approach takes into account complex behaviors and memory processes that usually go unrecognized by traditional integer order models during the transmission of diseases^24^. When there are two or more epidemics at once, then such a situation can be very difficult for public health systems. Modeling mathematics shows that the diseases affect each other. The current study employs a special sort of mathematical modeling to evaluate interventions in monkeypox and COVID-19 among high-risk populations^25^. For instance, it helps control diseases such as yellow fever, chikungunya, and HBV where vaccination and treatment are key factors^26^. In addition, these aspects may be incorporated into the epidemiological models through fractional frameworks so as to make them more realistic^27^. Unlike the other mpox outbreaks in recent years, this outbreak has largely spread through sexual contact. The target group had already a very high risk of acquiring HIV, it was primarily affected. Because it affected basically the same people, both diseases raised special health concerns because of this characteristic. Reductions in health focus were required so that sexual and social restrictions, such as those in that community, facilitate the spread of disease. Later this would help curb the outbreak and protect people from mpox and HIV^28^.

Consequently, the Caputo-Fabrizio fractional derivative was proposed after briefly looking at some basic properties of the exponential kernel used to model various engineering and physical processes^29^. Despite its exciting methods, there were some issues associated with the locality of this kernel that can serve to reduce its accuracy and applicability in certain areas^30^. To solve these problems, Atangana and Baleanu came up with the Atangana-Baleanu-Caputo (ABC) fractional derivative, which has the Mittag-Leffler function as a non-local, non-singular kernel^31^. The Caputo-Fabrizio fractional derivative has been associated with a new fractional integral which is defined using some exponential decay kernel such that it smoothens out singularities that are normally present in classical fractional calculus. In other words, this integral gives a more flexible and steady basis for system modeling in which there are memory and hereditary properties devoid of complications originating from singular kernels^32^. Analyzing the behavior of a model with memory effects requires an operator that extends differentiation for fractional orders. The model is based on a kernel, which is free from singularities and smooth. Thus, the solution is well-defined and stable unlike ones produced by classical fractional calculus with their singularities^33^.

The Atangana-Baleanu fractional derivative is a useful instrument for modeling complex systems that exhibit memory characteristics due to its non-singular and non-local kernel. Its unique features make it a prime candidate for stability and adaptability necessitating areas including higher level methods in image processing and so on^34^.The ABC fractional derivative has been employed in a variety of fields, such as epidemiology, where it more accurately depicts the spread of infectious diseases^35^. It also provides an insight into COVID-19 models through diffusions and dynamics control on infectious diseases for instance^36^. Moreover, the ABC derivative describes anomalous diffusion and viscoelastic materials better than ordinary methods used in physics and engineering for example^37^. To comprehend the systems through which various diseases spread and develop, advanced mathematical techniques facilitate the inclusion of historical information and the impacts of long-term memory^38,39^. In turn, researchers may use these fractional models in devising methods to stop or manage sicknesses, thus improving overall health of people who are impacted^40^. This study is thus driven by the desire for greater accuracy in predictive models that can guide public health initiatives and policy decisions in combating vector-borne diseases^41^.

Our studies focus on the dynamics of non-integer order and nonlocal, nonsingular kernels in modeling vector-borne diseases. The approach we have taken is a better representation of real-life dynamics because it incorporates fractional order calculus, which takes into account memory effects and long-range interactions more efficiently. Therefore, predictions about disease propagation and control are made more precisely using this methodology than is done with ordinary integer models. Furthermore, nonlocal and nonsingular kernel formation results in smoother and realistic output thus increasing the stability of the model when compared to others based on classical methods. The Atangana-Baleanu derivative is introduced, whose nonlocal and nonsingular kernels define the kernel for capturing spatial and temporal correlations in the transmission dynamics. The effects of past infections on current transmission dynamics are modelled by the kernels. This paper presents a fractional framework for vector-borne diseases, where a nonlocal and nonsingular kernel has been used to describe the complicated and non-integer patterns of disease transmission. The Atangana-Baleanu Caputo derivative is included in our model that gives a better and comprehensive representation of different relationships among vectors, their hosts and their environments that would lead to optimal prediction and understanding of epidemic trends.

The layout of this article: In section "Fractional-calculus preliminaries", we explain the fundamental results and ideas related to the AB fractional operator. Section "Evaluation of fractional dynamics" formulates a mathematical model of vector-borne disease infections incorporating vaccination. Section "Analysis of the model" gives an in-depth account of stability analysis, while section "Solution of the model" emphasizes on existence and uniqueness of solutions. A numerical scheme is constructed for the proposed model in section "Numerical scheme for the dynamics", while In section "Results and discussion" describes the results and discussion. Section "Conclusion" provides an extensive conclusion of the whole work.

## Fractional-calculus preliminaries

In this paper, we demonstrate the important theory related to the A-B operator comprising the Caputo derivative mentioned in literature^15^. This is also shown for the AB operator in reference^15^. All these outcomes and analyses were utilized for assessing the model.

### Definition 2.1

^15^. Suppose we define a function $l$ where, $l:[p,q]\rightarrow \mathbb {R}$ then according to this definition, the Caputo fractional derivative of order $\varepsilon$ with respect to $l$ can be written in terms of $p$:

$$\begin{aligned} ^{{C}}_{p}D^{\varepsilon }_{u}(l(u))= \frac{1}{\Gamma (s-\varepsilon )}\int _p^u l^s(\varsigma )(u-\varsigma )^{s-\varepsilon -1}d\varsigma . \end{aligned}$$

Let *s* belong to $\textbf{Z}$ and $\varepsilon$ belong to the interval $(s-1,s)$.

### Definition 2.2

^15^. Let’s assume there’s a function *l* such that for $q > p$, *l* belongs to the space $H^1(p, q)$ and $\varepsilon \in [0,1]$. The ABC operator fractional in Caputo representation is defined by:

$$\begin{aligned} ^{{ABC}}_{p}D^{\varepsilon }_{u}l(u)= \frac{B(\varepsilon )}{1-\varepsilon }\int _p^u l'(\varsigma )E_\varepsilon \bigg [-\varepsilon \frac{(u-\varsigma )^\varepsilon }{1-\varepsilon }\bigg ]d\varsigma . \end{aligned}$$

### Definition 2.3

^15^. let $^{ABC}_{p}I^{\varepsilon }_{t}l(u)$ where AB derivative integral can be described as:

$$\begin{aligned} ^{{ABC}}_{p}I^{\varepsilon }_{u}l(u)= \frac{1-\varepsilon }{B(\varepsilon )}l(u)+\frac{\varepsilon }{B(\varepsilon )\Gamma (\varepsilon )}\int _p^u l(\varsigma ) (u-\varsigma )^{\varepsilon -1}d\varsigma . \end{aligned}$$

It implies that the initial function is reachable with fractional-order $\varepsilon \rightarrow 0$.

### Theorem 2.4

^15^. For a function *l* defined on the interval $l \in C[m,n]$, then the following is true

$$\begin{aligned} \Vert ^{{ABC}}_{p}D^{\varepsilon }_{u}(l(u))\Vert <\frac{B(\varepsilon )}{1-\varepsilon }\Vert l(u)\Vert , \;where\; \Vert l(u)\Vert =max_{m\le u\le n}|l(u)|. \end{aligned}$$

In addition, the Lipschitz condition is satisfied by the ABC derivative as follows:

$$\begin{aligned} \Vert ^{{ABC}}_{p}D^{\varepsilon }_{u}l_1(u)- ^{{ABC}}_{p}D^{\varepsilon }_{u}l_2(u)\Vert <\mathcal {\varphi }_1 \Vert l_1(u)-l_2(u)\Vert . \end{aligned}$$

### Theorem 2.5

^15^. A unique solution of this fractional differential equation system

$$\begin{aligned} ^{{ABC}}_{p}D^{\varepsilon }_{t}l(t)=u(t), \end{aligned}$$

could potentially be expressed in the following manner:

$$\begin{aligned} {l(t)=\frac{1-\varepsilon }{B(\varepsilon )}u(t)+\frac{\varepsilon }{B(\varepsilon )\Gamma (\varepsilon )}\int _p^t u(\psi )(t-\psi )^{\varepsilon -1}d\psi .} \end{aligned}$$

##  Evaluation of fractional dynamics

In this section, we will define a mathematical model regarding how the transmission takes place in relation with vector as a means of conveying infection including vaccination and spraying of insecticides. The term $N_h$ will indicate humans’ population whilst $N_v$ refers to population of people who have structured diseases that lead to their inability for reproduction. We clearly delineate four classes of humans which are the susceptible $S_h$, those who were vaccinated $V_h$, those who contracted diseases $I_h$ and those who have recovered from certain infections $R_h$; on the other hand, vector is classified into two groups that include either susceptible or infected forms they are supposed to be represented as $S_v$ and $I_v$ respectively. In this expression, the recruitment rate for humans is represented by $\Lambda _h$, while the recruitment of vectors is denoted by $\Lambda _v$. The death rate for humans is indicated by $\mu _h$, and the death rate for vectors is represented by $\mu _v$. We assume that some amount *p* of the population vulnerable to infection get vaccinated against it and take their place among those who have received a shot of medication. The recovery rate from this infection will be represented as $\gamma$, disease-caused mortality will be denoted $\delta$, and vaccine efficacy will be referred to as $\nu$. A fraction *p* of the susceptible population is vaccinated and moves to the susceptible class. We assumed that the vaccination was not fully effective and moved to the susceptible class after losing the efficacy of the vaccine. Hence, our model with these aforementioned proposals can be described mathematically as follows:

1 $$\begin{aligned} \left\{ \begin{array}{rcl} \frac{d S_h}{dt} & =& \Lambda _h-\beta _h (1-r \epsilon ) \frac{S_h I_v}{N_h}-\mu _h S_h-pS_h+\omega V_h, \\ \frac{d V_h}{dt} & =& pS_h - \beta _h (1-r \epsilon )(1-\nu ) \frac{V_h I_v}{N_h}-\mu _h V_h-\omega V_h, \\ \frac{d I_h}{dt} & =& \beta _h (1-r \epsilon ) \frac{S_h I_v}{N_h}+\beta _h (1-r \epsilon )(1-\nu ) \frac{V_h I_v}{N_h}-(\mu _h+\gamma +\delta ) I_h, \\ \frac{d R_h}{dt} & =& \gamma I_h -\mu _h R_h, \\ \frac{dS_v}{dt} & =& \Lambda _v-\beta _v (1-r \epsilon ) \frac{S_v I_h}{N_h}-(\mu _v+\theta ) S_v, \\ \frac{d I_v}{dt} & =& \beta _v (1-r \epsilon ) \frac{S_v I_h}{N_h} - (\mu _h + \theta )I_v, \end{array}\right. \end{aligned}$$

where *r* is the rate of using treated bed nets and $\epsilon$ is the efficacy of bed nets which reduce the contact between susceptible and infected individuals of both the populations. In this formulation, $\theta$ is the rate at which insecticide spray reduces the population vectors. Here, we have the initial condition

2 $$\begin{aligned} \begin{array}{rcl} S_h(0)\ge 0,\ V_h(0)\ge 0,\ I_h(0)\ge 0,\ R_h(0)\ge 0,\ S_v(0)\ge 0,\ I_v(0)\ge 0. \end{array} \end{aligned}$$

In accordance with Model (1), all initial conditions for a system should be non-negative; thus, their representation is presented below:

$$S_h(0) = S_{h0}, V_h(0) = V_{h0}, I_h(0) = I_{h0}, R_h(0) = R_{h0}, S_v(0) = S_{v0}, I_v(0) = I_{v0}.$$

The model mentioned above (1) in fractional form can be expressed as:

3 $$\begin{aligned} \left\{ \begin{array}{rcl} ^{ABC}_0 D^{\upsilon }_t S_h & =& \Lambda _h-\beta _h (1-r \epsilon ) \frac{S_h I_v}{N_h}-\mu _h S_h-pS_h+\omega V_h, \\ ^{ABC}_0 D^{\upsilon }_t V_h & =& pS_h - \beta _h (1-r \epsilon )(1-\nu ) \frac{V_h I_v}{N_h}-\mu _h V_h-\omega V_h, \\ ^{ABC}_0 D^{\upsilon }_t I_h & =& \beta _h (1-r \epsilon ) \frac{S_h I_v}{N_h}+\beta _h (1-r \epsilon )(1-\nu ) \frac{V_h I_v}{N_h}-(\mu _h+\gamma +\delta ) I_h, \\ ^{ABC}_0 D^{\upsilon }_t R_h & =& \gamma I_h -\mu _h R_h, \\ ^{ABC}_0 D^{\upsilon }_t S_v & =& \Lambda _v-\beta _v (1-r \epsilon ) \frac{S_v I_h}{N_h}-(\mu _v +\theta ) S_v, \\ ^{ABC}_0 D^{\upsilon }_t I_v & =& \beta _v (1-r \epsilon ) \frac{S_v I_h}{N_h} - (\mu _h + \theta )I_v, \end{array}\right. \end{aligned}$$

where $0<\upsilon \le 1.$ In order to make epidemic modeling more efficacious in forecasting and controlling the diffusion of contagions. It is given that the Atangana-Baleanu derivative has a great capacity to represent its non-local nature as well as its non-singular behaviors, which can be important in describing some physical phenomena accurately. The atangana-Baleanu derivative is a mathematical structure that can easily morph into a model of memory systems with long-term relationships. It has a wide application area owing to its versatility.

### Theorem 3.1

The solutions of the proposed model (3) of vector-borne infection are non-negative and bounded.

## Analysis of the model

This part will emphasize the vector-born disease model with regard to *DFE*, $R_0$ and *LAS*. Let $\mathcal {E}_0$ represents the *DFE* obtained by taking the steady state of system (3) devoid of any infections. Fo this.

$$\mathcal {E}_0=\left( \frac{\Lambda _h (\mu _h + \omega )}{\mu _h (\mu _h + \omega + p)}, \frac{p \Lambda _h}{\mu _h (\mu _h + \omega + p)}, 0, 0, \frac{\Lambda _v}{\mu _v + \theta }, 0 \right) .$$

We presume that the basic reproduction number is denoted by $\mathcal {R}_0$ and various methods could be used to compute it. Our model’s $\mathcal {R}_0$ can be determined by following these steps:

$$\begin{aligned} \mathcal {F} = \begin{bmatrix} 0 & \beta _h (1-r \epsilon ) \frac{S_h^0}{N_h} + \beta _h (1-r \epsilon )(1-\nu ) \frac{V_h^0}{N_h} \\ \beta _v (1-r \epsilon ) \frac{S_v^0}{N_h} & 0 \end{bmatrix}, \mathcal {V} = \begin{bmatrix} \mu _h + \gamma + \delta & 0 \\ 0 & \mu _v + \theta \end{bmatrix}, \end{aligned}$$

and

$$\begin{aligned} \mathcal {V}^{-1} = \begin{bmatrix} \frac{1}{\mu _h + \gamma + \delta } & 0 \\ 0 & \frac{1}{\mu _v + \theta } \end{bmatrix}. \end{aligned}$$

From the above, we have $R_0$=$\mathcal {F} \mathcal {V}^{-1}$ as

$$\begin{aligned} \mathcal {F} \mathcal {V}^{-1} = \begin{bmatrix} 0 & \left( \beta _h (1-r \epsilon ) \frac{S_h^0}{N_h} + \beta _h (1-r \epsilon )(1-\nu ) \frac{V_h^0}{N_h}\right) \cdot \frac{1}{\mu _v + \theta } \\ \left( \beta _v (1-r \epsilon ) \frac{S_v^0}{N_h}\right) \cdot \frac{1}{\mu _h + \gamma + \delta } & 0 \end{bmatrix}. \end{aligned}$$

Putting DFE points, Then $\mathcal {R}_0$ is:

$$\begin{aligned} \mathcal {R}_0 = \sqrt{\frac{\beta _h \beta _v (1-r \epsilon )^2 \mu _v \left( (\mu _h + \omega ) + p (1-\nu ) \right) }{(\mu _h + \omega + p) (\mu _v + \theta )^2 (\mu _h + \gamma + \delta )}}. \end{aligned}$$

The threshold parameter $\mathcal {R}_0$ is determined using the next-generation matrix approach. An explicit expression for $\mathcal {R}_0$ is derived within the framework of the Atangana-Baleanu derivative, and the stability of the disease-free equilibrium is analyzed in this context.

### Theorem 4.1

If $\mathcal {R}_0$ is less than 1, then the steady-state $\mathcal {E}_0$ locally asymptotically stable, otherwise unstable.

### Proof

In order to attain the stability outcome that is desired, we consider the Jacobian matrix at $\mathcal {E}_0$ which is given by

$$\begin{aligned} \mathcal {J}(E_0)= \begin{bmatrix} -(\mu _h + p) & \omega & 0 & 0 & 0 & -\beta _h (1-r \epsilon ) \frac{S_h^0}{N_h} \\ p & -(\mu _h + \omega ) & 0 & 0 & 0 & -\beta _h (1-r \epsilon )(1-\nu ) \frac{V_h^0}{N_h} \\ 0 & 0 & -(\mu _h + \gamma + \delta ) & 0 & 0 & \beta _h (1-r \epsilon ) \frac{S_h^0}{N_h} + \beta _h (1-r \epsilon )(1-\nu ) \frac{V_h^0}{N_h} \\ 0 & 0 & \gamma & -\mu _h & 0 & 0 \\ 0 & 0 & -\beta _v (1-r \epsilon ) \frac{S_v^0}{N_h} & 0 & -(\mu _v + \theta ) & 0 \\ 0 & 0 & \beta _v (1-r \epsilon ) \frac{S_v^0}{N_h} & 0 & 0 & -(\mu _v + \theta ) \end{bmatrix}. \end{aligned}$$

It is clear that the eigenvalues of $\mathcal {J}(E_0)$ are negative hence giving us the desired results. Thus we define the characteristic equation as follows:

$$\begin{aligned} & \det [\mathcal {J}(E_0)-\chi I]=0,\\ & \begin{vmatrix} -(\mu _h + p)-\chi&\omega&0&0&0&-\beta _h (1-r \epsilon ) \frac{S_h^0}{N_h} \\ p&-(\mu _h + \omega )-\chi&0&0&0&-\beta _h (1-r \epsilon )(1-\nu ) \frac{V_h^0}{N_h} \\ 0&0&-(\mu _h + \gamma + \delta )-\chi&0&0&\beta _h (1-r \epsilon ) \frac{S_h^0}{N_h} + \beta _h (1-r \epsilon )(1-\nu ) \frac{V_h^0}{N_h} \\ 0&0&\gamma&-\mu _h-\chi&0&0 \\ 0&0&-\beta _v (1-r \epsilon ) \frac{S_v^0}{N_h}&0&-(\mu _v + \theta )-\chi&0 \\ 0&0&\beta _v (1-r \epsilon ) \frac{S_v^0}{N_h}&0&0&-(\mu _v + \theta )-\chi \end{vmatrix}=0 \end{aligned}$$

Based on the information provided, As like the $5^{th}$ and $6^{th}$ eigenvalues are negative, we reduce the $\mathcal {J}(E_0)$ to the sub-matrix formed:

$$\begin{aligned} \begin{vmatrix} -(\mu _h + p) - \chi&\omega&0&0 \\ p&-(\mu _h + \omega ) - \chi&0&0 \\ 0&0&-(\mu _h + \gamma + \delta ) - \chi&0 \\ 0&0&\gamma&-\mu _h - \chi \end{vmatrix}=0. \end{aligned}$$

$3^{rd}$ and $4^{th}$ eigenvalues are also negative, therefore

$$\begin{aligned} \mathcal {J}(E_1)= \begin{bmatrix} -(\mu _h + p) & \omega \\ p & -(\mu _h + \omega ) \end{bmatrix}, \end{aligned}$$

as $\text {Tr}(\mathcal {J}(E_1))< 0$ and $\text {det}(\mathcal {J}(E_1))> 0$), For this $R_0 < 1$, so the system 3 is *LAS* for DFE. $\square$

Here, we determined the local stability of DFE of our system, which shows that the diseases dies out for $\mathcal {R}_0<1$.

## Solution of the model

In this paper we use the fixed-point theory to prove the existence and uniqueness of solutions in our model. It presents a proper theory to analyze the behavior of vector borne disease model. The above system describes a vector carried sickness with A-B derivative as follows:

4 $$\begin{aligned} \left\{ \begin{array}{rcl} ^{ABC}_0 D^{\upsilon }_t S_h & =& \Lambda _h-\beta _h (1-r \epsilon ) \frac{S_h I_v}{N_h}-\mu _h S_h-pS_h+\omega V_h, \\ ^{ABC}_0 D^{\upsilon }_t V_h & =& pS_h - \beta _h (1-r \epsilon )(1-\nu ) \frac{V_h I_v}{N_h}-\mu _h V_h-\omega V_h, \\ ^{ABC}_0 D^{\upsilon }_t I_h & =& \beta _h (1-r \epsilon ) \frac{S_h I_v}{N_h}+\beta _h (1-r \epsilon )(1-\nu ) \frac{V_h I_v}{N_h}-(\mu _h+\gamma +\delta ) I_h, \\ ^{ABC}_0 D^{\upsilon }_t R_h & =& \gamma I_h -\mu _h R_h, \\ ^{ABC}_0 D^{\upsilon }_t S_v & =& \Lambda _v-\beta _v (1-r \epsilon ) \frac{S_v I_h}{N_h}-(\mu _h + \theta ) S_v, \\ ^{ABC}_0 D^{\upsilon }_t I_v & =& \beta _v (1-r \epsilon ) \frac{S_v I_h}{N_h} - (\mu _h + \theta ) I_v, \end{array}\right. \end{aligned}$$

this can be further expressed as follows:

5 $$\begin{aligned} \begin{array}{ll} ^{{ABC}}_{0} D^{\upsilon }_{t}w(t)=\mathcal {J}(t,w(t)),\\ w(0)=w_0, \;\; 0<t<\mathcal {T}<\infty , \end{array} \end{aligned}$$

$w(t)=(S_h,V_h,I_h,R_h,S_v,R_v)$ is our state variable in this context. In addition, $\mathcal {J}$ is continuous function. To elaborate more;

$$\begin{aligned} \mathcal {J}=\left( \begin{array}{cccccc} \mathcal {J}_1\\ \mathcal {J}_2\\ \mathcal {J}_3\\ \mathcal {J}_4\\ \mathcal {J}_5\\ \mathcal {J}_6 \end{array} \right) =\left( \begin{array}{cccccc} \Lambda _h-\beta _h (1-r \epsilon ) \frac{S_h I_v}{N_h}-\mu _h S_h-p S_h+\omega V_h, \\ pS_h - \beta _h (1-r \epsilon )(1-\nu ) \frac{V_h I_v}{N_h}-\mu _h V_h-\omega V_h,\\ \beta _h (1-r \epsilon ) \frac{S_h I_v}{N_h}+\beta _h (1-r \epsilon )(1-\nu ) \frac{V_h I_v}{N_h}-(\mu _h+\gamma +\delta ) I_h, \\ \gamma I_h -\mu _h R_h, \\ \Lambda _v-\beta _v (1-r \epsilon ) \frac{S_v I_h}{N_h}-(\mu _h + \theta ) S_v,\\ \beta _v (1-r \epsilon ) \frac{S_v I_h}{N_h} - (\mu _h + \theta )I_v, \end{array} \right) . \end{aligned}$$

when appropriate initial conditions like $w_0(t)=(S_h(0),V_h(0),I_h(0),R_h(0),S_v(0),I_v(0))$ were defined. More so, it possesses the Lipschitz property as follows:

6 $$\begin{aligned} \Vert \mathcal {J}(t, l_1(t))-\mathcal {J}(t, l_2(t))\Vert\le & \textrm{W}\Vert w_1(t)-w_2(t)\Vert . \end{aligned}$$

The existence and uniqueness of system (4) will be analysed in the next result.

### Theorem 5.1

If this condition holds, there exists a unique solution for the proposed system (4) involving vector-borne illness.

7 $$\begin{aligned} \frac{(1-\varepsilon )}{ABC(\varepsilon )}\mathcal {V}+\frac{\varepsilon }{ABC(\varepsilon )\Gamma (\varepsilon )}\mathcal {T}^{\varepsilon }_{max}\mathcal {V}<1. \end{aligned}$$

### Proof

To get the solution, we employ the A-B fractional integral in equation (2.3) on the system in equation (5) resulting in:

8 $$\begin{aligned} w(t)=w_0+\frac{1-\upsilon }{ABC(\varepsilon )}\mathcal {J}(t,w(t))+\frac{\varepsilon }{ABC(\varepsilon )\Gamma (\varepsilon )}\int _0^t(t-\varpi )^{\varepsilon -1}\mathcal {J} (\varpi ,w(\varpi ))d\varpi . \end{aligned}$$

The same interval $I$ needs to be taken as $(0,\mathcal {T})$, and the operator $T$ is represented as follows: $\Lambda :\mathcal {P}(I,\textrm{R}^6)\rightarrow \mathcal {P}(I,\textrm{R}^6)$

9 $$\begin{aligned} \Lambda [w(u)]=w_0+\frac{1-\varepsilon }{ABC(\varepsilon )}\mathcal {J}(u,w(u))+\frac{\varepsilon }{ABC(\varepsilon )\Gamma (\varepsilon )}\int _0^u(u-\varpi )^{\varepsilon -1}\mathcal {J} (\varpi ,w(\varpi ))d\varpi , \end{aligned}$$

subsequently, we have equation (8) such as:

10 $$\begin{aligned} w(u)=\Lambda [w(u)]. \end{aligned}$$

For an arbitrary set *I*, we denote its supreme norm by $\Vert .\Vert _I$, which is denoted mathematically as follows.

11 $$\begin{aligned} \Vert w(u)\Vert _I=\sup _{u\in I}\Vert w(u)\Vert ,\;\;w(u)\in \mathcal {P}. \end{aligned}$$

According to the fact that $\mathcal {P}(I,\textrm{R}^6)$ can be classified as a Banach space given the existence of norms $\Vert .\Vert _I$. In addition, it is evident that

12 $$\begin{aligned} \bigg \Vert \int _0^u \mathcal {K} (u,\varpi )w(\varpi )d\varpi \bigg \Vert \le \mathcal {T}\Vert \mathcal {K} (u,\varpi )\Vert _I \Vert w(u)\Vert _I. \end{aligned}$$

$\mathcal {P}(I,\textrm{R}^6)$ and $\mathcal {P}(I^2,\textrm{R})$ respectively are places where both *w*(*u*) and $\mathcal {K}(u,\varpi )$ belong. The situation is such that

13 $$\begin{aligned} \Vert \mathcal {K}(u,\varpi )\Vert _I=\sup _{u,\varpi \in I}|\mathcal {K}(u,\varpi )|. \end{aligned}$$

Employing the definition of $\Lambda$ as given in (10), we get the below outcomes:

14 $$\begin{aligned} \Vert \Lambda [w_1(u)]- \Lambda [w_2(u)]\Vert _I\le & \bigg \Vert \frac{(1-\varepsilon )}{ABC(\varepsilon )} (\mathcal {J} (u,w_1(u))-\mathcal {J}(u,w_2(u))+ \frac{\varepsilon }{ABC(\varepsilon )\Gamma (\varepsilon )} \nonumber \\ & \times \int ^u_0(u-\varpi )^{\varepsilon -1}(\mathcal {J}(\varpi ,w_1(\varpi ))- \mathcal {J}(\varpi ,w_2(\varpi )))d\varpi \bigg \Vert _I. \end{aligned}$$

Additionally, as a result of employing the Lipschitz restriction (6), together with the result from equation (1), we get that

15 $$\begin{aligned} \Vert \Lambda [w_1(u)]- \Lambda [w_1(u)]\Vert _I\le & \bigg [\frac{(1-\varepsilon )\mathcal {V}}{ABC(\varepsilon )}+\frac{\varepsilon }{ABC(\varepsilon )\Gamma (\varepsilon )} \mathcal {V}\mathcal {T}^{\varepsilon }_{max}\bigg ]\Vert w_1(t)-w_2(t)\Vert _I. \end{aligned}$$

As a result, we get the following

16 $$\begin{aligned} \Vert \Lambda [w_1(u)]-\Lambda [w_1(u)]\Vert _I\le & \textrm{D}\Vert w_1(u)-w_2(u)\Vert _I. \end{aligned}$$

where

$$\begin{aligned} \textrm{D}=\frac{(1-\varepsilon )\mathcal {V}}{ABC(\varepsilon )}+\frac{\varepsilon }{ABC(\varepsilon )\Gamma (\varepsilon )}\mathcal {V}\mathcal {T}^{\varepsilon }_{max}. \end{aligned}$$

It is evident that in the event when the previously stated condition (7) is satisfied, $\Lambda$ becomes a contraction. As a result, it follows that the vector-borne infection model, described by system (4), has a unique solution. $\square$

The solution of our model is investigated with the help of fixed-point theory in the context of the fractional ABC derivative.

## Numerical scheme for the dynamics

Here, we are to solve our system (4) numerically. To this end, we first discuss the Atangana-Baleanu derivative, which is stated as follows.

17 $$\begin{aligned} ^{ABC}_0 D^{\varepsilon }_u f(u)= g(u,f(u)), \end{aligned}$$

the equation that precedes it should be converted into another one as per the instructions given in^16^:

18 $$\begin{aligned} f(u)-f(0)= \frac{1- \varepsilon }{AB(\varepsilon )} g(u,f(u))+ \frac{\varepsilon }{AB(\varepsilon ) \Gamma (\varepsilon )} \int _0^u g(\theta ,f(\theta )) (u-\theta )^{(\varepsilon -1)} d\theta . \end{aligned}$$

The aforementioned towards $u_{s+1}=(s+1)\Delta u$ might be expressed as

19 $$\begin{aligned} f(u_{s+1})-f(0)= \frac{1- \varepsilon }{AB(\varepsilon )} g(u_s,f(u_s))+ \frac{\varepsilon }{AB(\varepsilon ) \Gamma (\varepsilon )} \int _0^{u_{s+1}} g(\theta ,f(\theta )) (u_{s+1}-\theta )^{(\varepsilon -1)} d\theta . \end{aligned}$$

this can be converted into:

20 $$\begin{aligned} f(u_{s+1})= f(0)+ \frac{1- \varepsilon }{AB(\varepsilon )} g(u_s,f(u_s))+ \frac{\varepsilon }{AB(\varepsilon ) \Gamma (\varepsilon )} \Sigma _{\imath =2}^{s} \int _{u_\imath }^{u_{\imath +1}} g(\theta ,f(\theta )) d\theta . \end{aligned}$$

In this next step, the Newton polynomial method is utilized for approximating *g*(*u*, *f*(*u*)) as follows.

21 $$\begin{aligned} P_u (\theta )= & g(u_{s-2}, f(u_{s-2})) + \frac{g(u_{s-1}, f(u_{s-1})) - g(u_{s-2}, f(u_{s-2}))}{\Delta u} (\theta - u_{s-2}) \nonumber \\ & + \frac{g(u_{s}, f(u_{s})) - 2g(u_{s-1}, f(u_{s-1})) + g(u_{s-2}, f(u_{s-2}))}{2(\Delta u)^2} (\theta - u_{s-2})(\theta - u_{s-1}). \end{aligned}$$

If we involve the polynomial that has been mentioned earlier in (20), then we can derive:

22 $$\begin{aligned} f^{s+1}= & f^0 + \frac{1 - \varepsilon }{AB(\varepsilon )} g(u_s, f(u_s)) + \frac{\varepsilon }{AB(\varepsilon ) \Gamma (\varepsilon )} \sum _{\imath =2}^{s} \int _{u_\imath }^{u_{\imath +1}} \bigg ( g(u_{\imath -2}, f^{\imath -2}) \nonumber \\ & + \frac{g(u_{\imath -1}, f^{\imath -1}) - g(u_{\imath -2}, f^{\imath -2})}{\Delta u} (\theta - u_{\imath -2}) \nonumber \\ & + \frac{g(u_{\imath }, f^{\imath }) - 2g(u_{\imath -1}, f^{\imath -1}) + g(u_{\imath -2}, f^{\imath -2})}{2 (\Delta u)^2} (\theta - u_{\imath -2})(\theta - u_{\imath -1}) \bigg ) \nonumber \\ & \times (u_{s+1} - \theta )^{\varepsilon -1} d\theta , \end{aligned}$$

moreover, we get

23 $$\begin{aligned} f^{s+1}= & f^0 + \frac{1 - \varepsilon }{AB(\varepsilon )} g(u_s, f(u_s)) + \frac{\varepsilon }{AB(\varepsilon ) \Gamma (\varepsilon )} \sum _{\imath =2}^{s} \bigg ( \nonumber \\ & \int _{u_\imath }^{u_{\imath +1}} g(u_{\imath -2}, f^{\imath -2}) (u_{s+1} - \theta )^{\varepsilon -1} d\theta \nonumber \\ & + \int _{u_\imath }^{u_{\imath +1}} \frac{g(u_{\imath -1}, f^{\imath -1}) - g(u_{\imath -2}, f^{\imath -2})}{\Delta u} (\theta - u_{\imath -2}) (u_{s+1} - \theta )^{\varepsilon -1} d\theta \nonumber \\ & + \int _{u_\imath }^{u_{\imath +1}} \frac{g(u_{\imath }, f^{\imath }) - 2g(u_{\imath -1}, f^{\imath -1}) + g(u_{\imath -2}, f^{\imath -2})}{2 (\Delta u)^2} \nonumber \\ & \quad (\theta - u_{\imath -2})(\theta - u_{\imath -1}) (u_{s+1} - \theta )^{\varepsilon -1} d\theta \bigg ). \end{aligned}$$

Through simplification, the following result is achieved:

24 $$\begin{aligned} f^{s+1}= & f^0+ \frac{1-\varepsilon }{AB(\varepsilon )}g(u_s,x(u_s)) + \frac{\varepsilon }{AB(\varepsilon ) \Gamma (\varepsilon )} \sum ^{s}_{\imath =2} g (u_{\imath -2},f^{\imath -2}) \Delta u \int _{u_\imath }^{u_{\imath +1}} (u_{s+1}-\theta )^{\varepsilon -1} d\theta \nonumber \\ & +\frac{\varepsilon }{AB(\varepsilon ) \Gamma (\varepsilon )} \sum ^{s}_{\imath =2} \frac{g (u_{\imath -1},f^{\imath -1})-g (u_{\imath -2},f^{\imath -2})}{\Delta u} \int _{u_\imath }^{u_{\imath +1}} (\theta -u_{\imath -2}) (u_{s+1}-\theta )^{\varepsilon -1} d\theta \nonumber \\ & +\frac{1}{\Gamma (\varepsilon )} \sum ^{s}_{\imath =2} \frac{g (u_{\imath },f^{\imath })-2g (u_{\imath -1},f^{\imath -1})+g (u_{\imath -2},f^{\imath -2})}{2 (\Delta u)^2} \nonumber \\ & \times \int _{u_\imath }^{u_{\imath +1}} (\theta -u_{\imath -2})(\theta -u{\imath -1}) (u_{s+1}-\theta )^{\varepsilon -1} d \theta , \end{aligned}$$

The method described below can be used to compute the integrals mentioned above.

25 $$\begin{aligned} \int _{u_\imath }^{u_{\imath +1}} (u_{s+1}-\theta )^{\varepsilon -1} d \theta= & \frac{(\Delta u)^\varepsilon }{\varepsilon } \bigg ( (s-\imath +1)^\varepsilon -(s-\imath )^\varepsilon \bigg ) \nonumber \\ \int _{u_\imath }^{u_{\imath +1}} (\theta -u_{\imath -2}) (u_{s+1}-\theta )^{\varepsilon -1} d \theta= & \frac{(\Delta u)^{\varepsilon +1}}{\varepsilon (\varepsilon +1)} \bigg ( (s-\imath +1)^\varepsilon (s-\imath +3+2 \varepsilon ) \nonumber \\ & - (s-\imath )^\varepsilon (s-\imath +3+3 \varepsilon ) \bigg ) \nonumber \\ \int _{u_\imath }^{u_{\imath +1}} (\theta -u_{\imath -2}) (\theta -u_{\imath -1}) (u_{s+1}-\theta )^{\varepsilon -1} d \theta= & \frac{(\Delta u)^{\varepsilon +2}}{\varepsilon (\varepsilon +1)(\varepsilon +2)} \times \nonumber \\ & \bigg [(s-\imath +1)^\varepsilon V_1 -(r-\imath )^\varepsilon V_2 \bigg ]. \end{aligned}$$

So, $V_1=2(s-\imath )^2+(3\varepsilon +10) (r-\imath )+2\varepsilon ^2+9 \varepsilon +12,$ and $V_2=2(s-\imath )^2+(5\varepsilon +10) (s-\imath )+6\varepsilon ^2+18 \varepsilon +12$. We obtain after simplification:

26 $$\begin{aligned} f^{s+1}= & f^0+ \frac{1-\varepsilon }{AB(\varepsilon )} g (u_s,f(u_s)) \nonumber \\ & +\frac{\varepsilon (\Delta u)^\varepsilon }{AB(\varepsilon )\Gamma (\varepsilon +1)} \sum ^{s}_{\imath =2} g (u_{\imath -2},f^{\imath -2}) [(s-\imath +1)^\varepsilon - (s-\imath )^\varepsilon ] \nonumber \\ & +\frac{\varepsilon (\Delta u)^\varepsilon }{AB(\varepsilon )\Gamma (\varepsilon +2)} \sum ^{s}_{\imath =2} [g (u_{\imath -1},f^{\imath -1}) - g (u_{\imath -2},f^{\imath -2}) ] \nonumber \\ & \times \bigg ( (s-\imath +1)^\varepsilon (s-\imath +3+2 \varepsilon ) - (s-\imath )^\varepsilon (s-\imath +3+3 \varepsilon ) \bigg ) \nonumber \\ & +\frac{\varepsilon (\Delta u)^\varepsilon }{2AB(\varepsilon )\Gamma (\varepsilon +3)} \sum ^{r}_{\imath =2} [f (u_{\imath },f^{\imath })-2g (u_{\imath -1},f^{\imath -1}) + g (u_{\imath -2},f^{\imath -2}) ] \nonumber \\ & \times \bigg [(s-\imath +1)^\varepsilon V_1 -(s-\imath )^\varepsilon V_2 \bigg ]. \end{aligned}$$

The Atangana-Baleanu-Caputo (ABC) fractional derivative is a robust mathematical tool for capturing memory and nonlocal effects in epidemic models. However, it has several limitations that warrant consideration. The nonlocal nature of the ABC derivative increases computational complexity, making simulations more resource-intensive, while the derivation of analytical solutions is often nontrivial. The accuracy of models incorporating the ABC derivative is highly sensitive to precise parameter calibration, with misestimations potentially affecting stability and reliability. Furthermore, the physical interpretation of the ABC derivative in the context of epidemiology remains less well-established, and its applicability is constrained in systems exhibiting abrupt changes, such as those caused by sudden interventions or shifts in transmission dynamics. Despite these challenges, the ABC derivative remains a valuable framework when paired with high-quality data and a clear understanding of its underlying assumptions and limitations.

## Results and discussion

The proposed infectious model time series will be depicted using the above approach. Monitoring, controlling, and understanding infectious diseases primarily involves time series analysis. It gives an insight into how they spread with time, warns before they could actually spread and measures up well the effectiveness of measures taken. This type of information can better inform specific public health measures. We showed the impact of input factors on the output of the proposed system. The subsequent illustrations indicate that certain parameters influence the process of infection.

**Figure 1:**
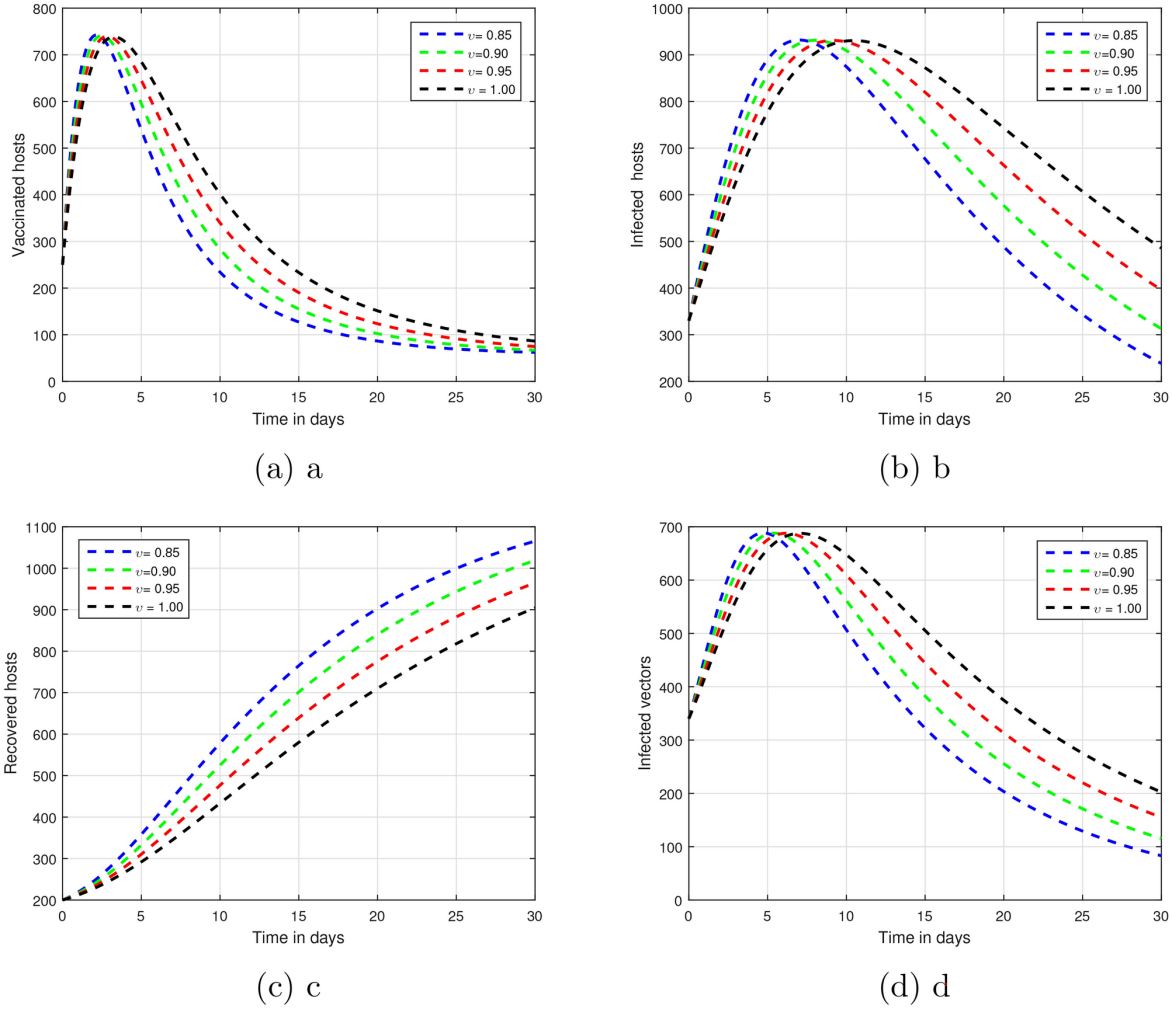
A time series evaluation was conducted on the proposed vector-borne infection model with the fluctuating input parameter $\upsilon$, that is, $\upsilon =0.85, 0.90, 0.95, 1.00$.

**Figure 2:**
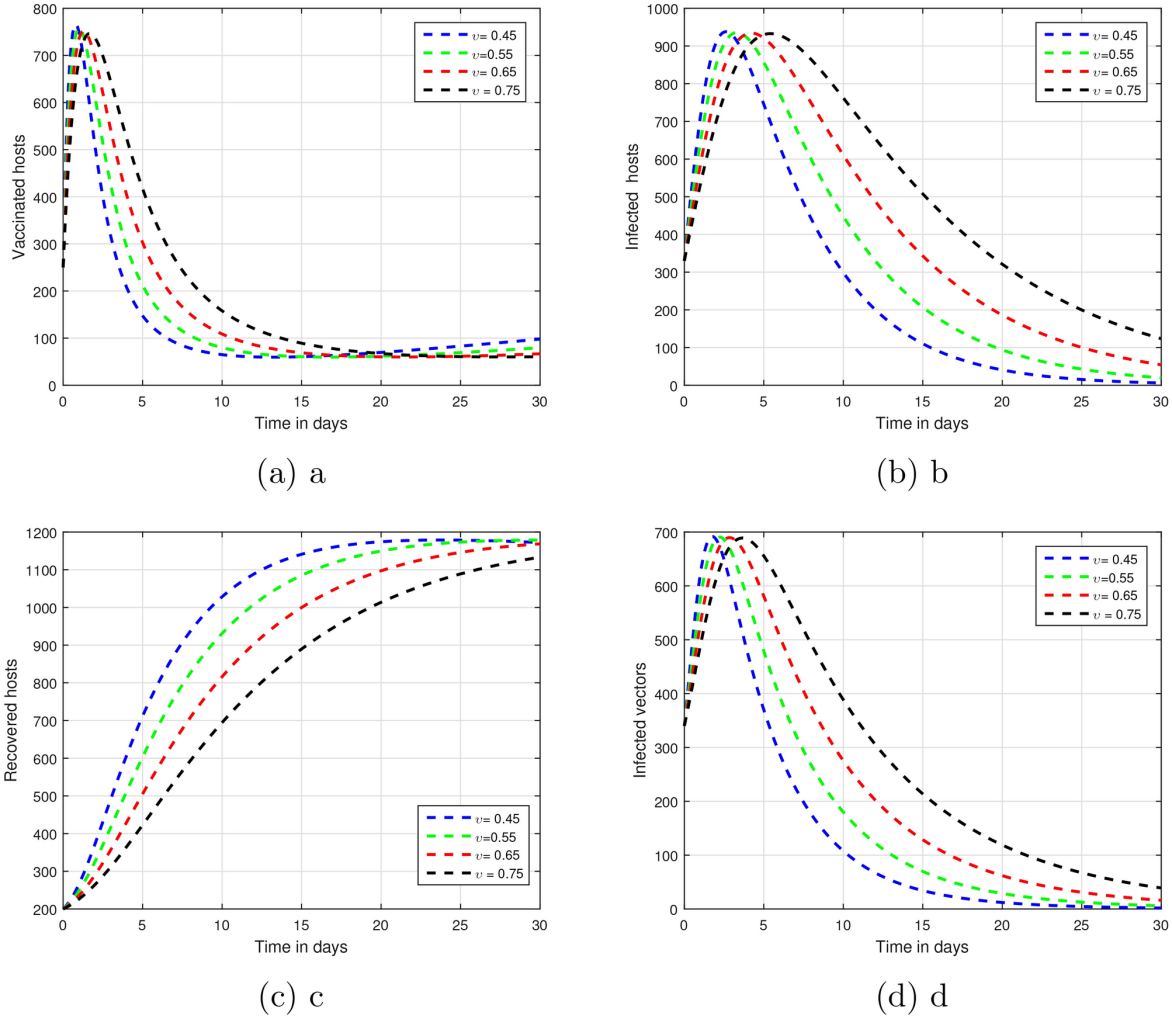
In the suggested system of vector-borne illness with the changing input parameter $\upsilon$, that is $\upsilon = 0.5, 0.6, 0.7$ and 0.8 an evaluation on time series has been conducted.

**Figure 3:**
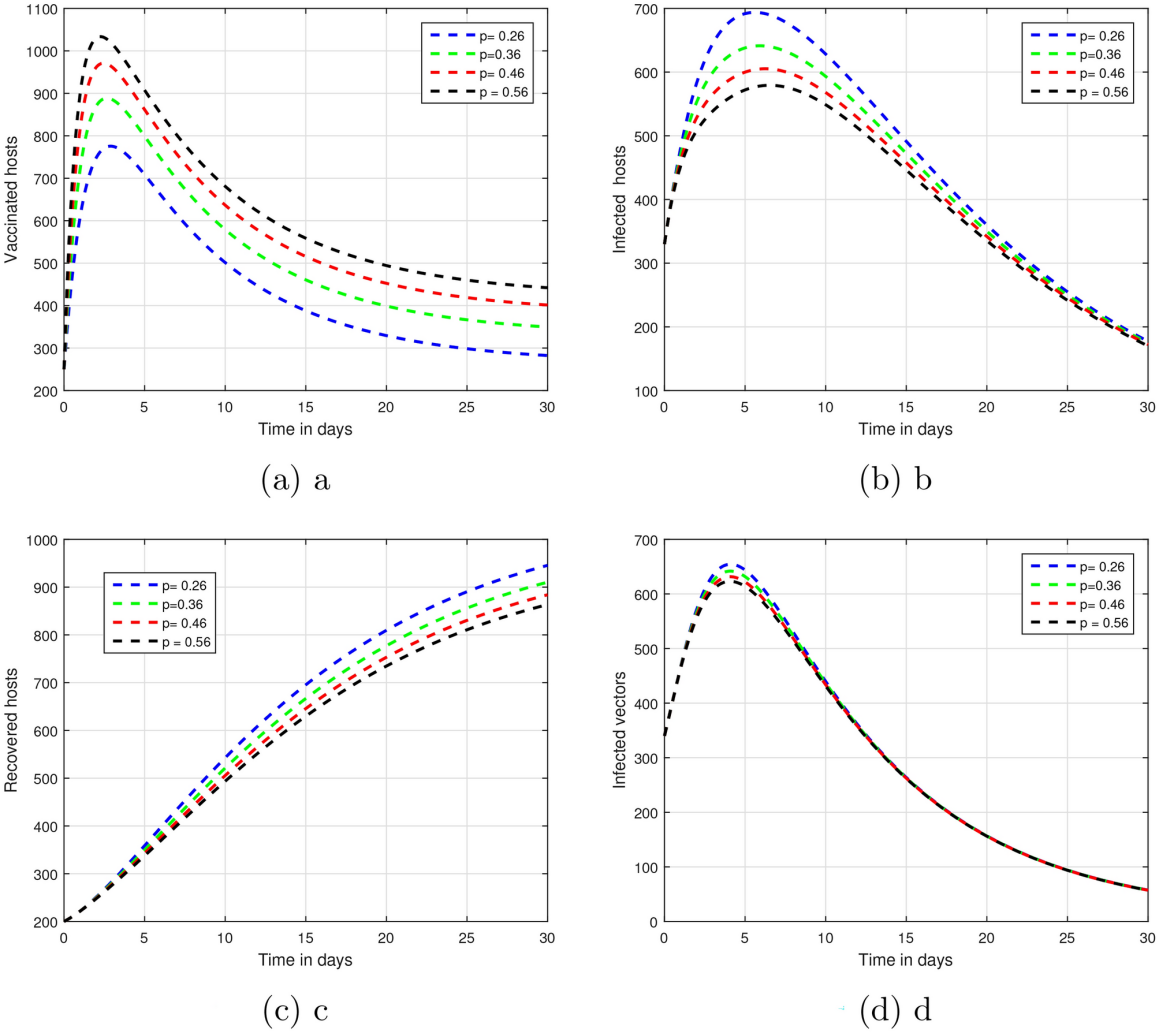
Graphical views of the dynamical behavior of our model of vector-borne infections based on various input factor values $\beta$, that is $\beta = 0.20, 0.40, 0.60, 0.80.$.

**Figure 4:**
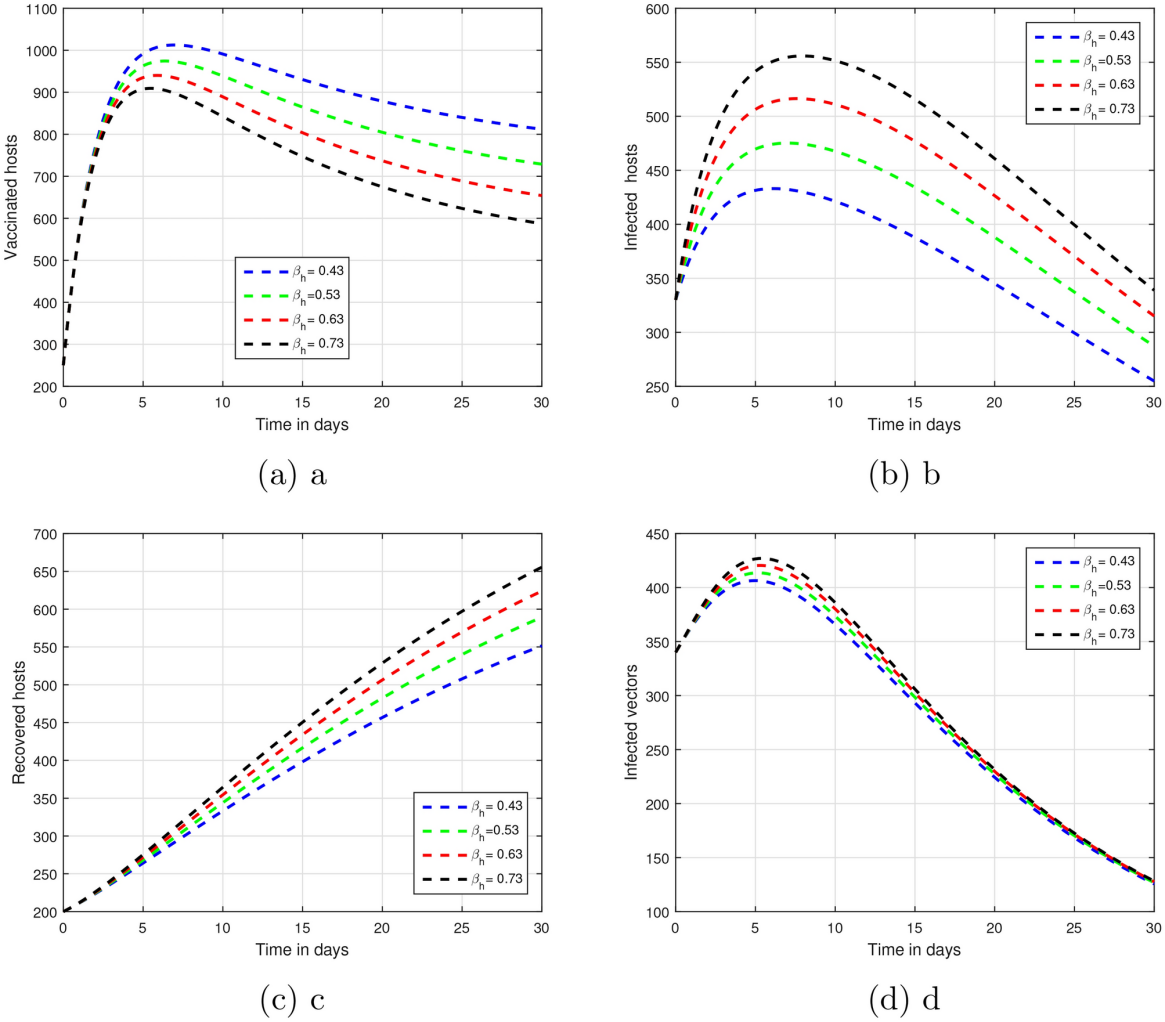
We present here graphical views of the dynamical behavior of our model of vector-borne infections based on various input factor values $\beta$, that is $\beta = 0.45, 0.55, 0.65, 0.75$.

**Figure 5:**
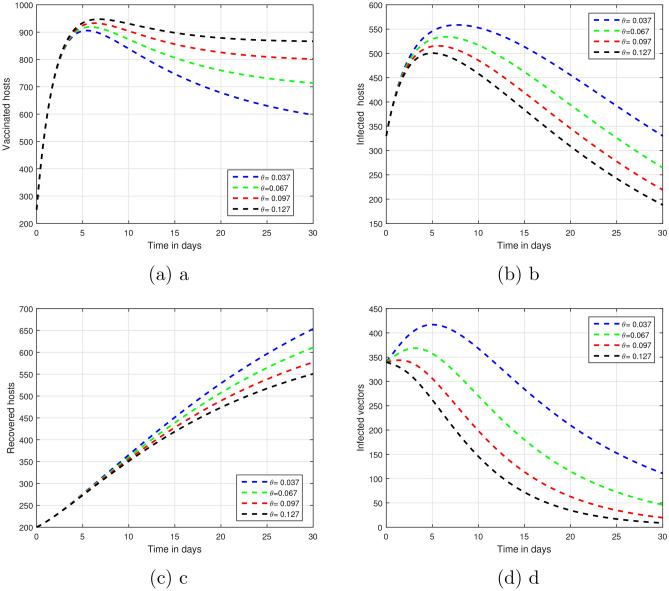
When $\theta$ takes values of $\theta =0.20,0.30,0.40,0.50$, show how the disease transmission model for vector-borne diseases will behave.

- The fractional parameter $\upsilon$ is analysed in the first simulation presented in Figs. 1 and 2 with regards to its impact on vector-borne disease transmission over time.
- The values of $\upsilon$ were set 1.00, 0.95, 0.90, and 0.85 in Fig. 1.
- As depicted in Fig. 2, values of $\upsilon$ are ; 0.45, 0.55, 0.65 and 0.75.
- A thorough understanding of a system response in different situations can be obtained through systematic investigation of those fractional values.
- Infection dynamics are significantly affected by the fractional parameter $\upsilon$.
- Among the methods for controlling epidemics, $\upsilon$ seems to be the most effective.
- Public authorities should prioritize research on $\upsilon$ to better understand and potentially reduce disease impact.
- The input parameter *p* has an effect on the dynamics of vector-borne infections as shown in Fig. 3.
- While infection levels can be reduced by *p*, it is advisable to increase vaccination efficacy for improved control.
- The simulation used $\rho$ values of 0.26, 0.36, 0.46, and 0.56.
- Biologically impact are seen Changing input parameters as seen in the Figs. 4 and 5.
- For different values of $\beta _h$ being 0.43, 0.53, 0.63, and 0.73 shown in Fig. 4 while $\theta$ set at the same time was 0.037, 0.067, 0.097, and 0.127 in Fig. 5.
- A detailed study of that which factors affect asymptomatic and infected hosts is performed.
- Insight from these results is important so that public health and intervention strategies can be effectively designed.
- In order to develop successful control measures for the transmission of infections via vectors it is important to comprehend how these variables interact among themselves.

The input parameters were chosen based on reasonable values found in existing research on vector-borne diseases. These parameters are used to show that the model works and to study that changes in important factors, like transmission rates and vaccination coverage, affect the results. As such, the results indicative the significance and sensitivity of the model to certain key parameters, as well as their effects on the observed dynamic features of disease transmission. The results show that $R_0$ is most sensitive to changes in vaccination rates.

## Conclusion

Vector-borne infections posed a significant challenge to global health systems and economies due to their widespread impact and the considerable resources required for prevention, control, and treatment. In this study, a mathematical model was formulated to describe the transmission dynamics of a vector-borne infection, incorporating the effect of vaccination through the Atangana-Baleanu derivative. The model’s solutions were shown to be positive and bounded for positive initial values of the state variable. The fundamental concepts and theoretical framework of fractional calculus were introduced to facilitate the model’s analysis. The threshold parameter, denoted by $\mathcal {R}_0$, was derived using the next-generation matrix method, and the local asymptotic stability of the system at the disease-free equilibrium was investigated. Fixed-point theory was employed to establish the existence of solutions for the proposed model. Additionally, a numerical scheme was developed to examine the system’s dynamical behavior under varying input parameters. Numerical simulations were performed to demonstrate the influence of these parameters on the model’s dynamics. The findings underscored critical factors affecting the transmission and control of vector-borne infections, providing valuable insights into strategies for their prevention and mitigation.

Future studies will investigate the impact of pulse immunization on the dynamics of vector-borne infections. Additionally, the individual effects of these immunization strategies will be examined, and the transmission dynamics of vector-borne infections will be incorporated within a stochastic framework.

## Data Availability

The data that support the findings of the study are available from the corresponding author upon reasonable request.
